# Embryo Biopsy Can Offer More Information Than Just Ploidy Status

**DOI:** 10.3389/fcell.2020.00078

**Published:** 2020-02-12

**Authors:** Arnav Lal, William E. Roudebush, Renee J. Chosed

**Affiliations:** Department of Biomedical Sciences, University of South Carolina School of Medicine Greenville, Greenville, SC, United States

**Keywords:** cell-free DNA, apoptosis, blastocoel fluid, mRNA, preimplantation genetic testing for aneuploidies, embryo selection, aneuploidy, implantation potential

## Abstract

As a byproduct of increasing infertility cases, the use of medically assisted reproduction (MAR) has increased. As such, the need to gain information regarding the implantation potential of specific MAR preimplantation embryos prior to transfer has become increasingly critical. One potential source of this information is contained in the blastocoel fluid from day 5/6 embryos. This fluid contains cell-free DNA, proteins, RNA, metabolites, exosomes, etc., and analysis of these contents provides clinicians with an opportunity to gain more data regarding potential of each embryo. While application of preimplantation genetic testing for aneuploidies (PGT-A) may be limited to women of advanced maternal age or with recurrent pregnancy loss, the fluid taken at the time of embryo biopsy can be analyzed for any frozen embryo as well as PGT-A embryos. In both cases, blastocoel fluid analysis provides information regarding a preimplantation embryo’s potential for implantation. Moreover, as remnants of apoptosis, embryonic cell-free DNA (cfDNA) and mRNA may lead clinicians to better understand and predict the extent of self-correction occurring within the preimplantation embryo. While analysis of blastocoel components are not yet viable replacements for PGT-A, their study may still reveal critical clinical information about the implantation potential for any given embryo.

## Introduction

### Infertility and IVF

Infertility is an increasingly prevalent condition which characterizes the inability of a couple to conceive a child. Unfortunately, infertility has become increasingly stigmatized and oftentimes leads to discrimination, depression, and ostracism for patients and their spouses (Cui, 2010). Factors such as changing cultural norms (which push pregnancies to later ages), an increase in obesity, smoking, alcohol and drug use, as well as environmental pollution all play parts in lowering fertility rates; numbers of infertile individuals have quickly risen past 15% of reproductive age couples (Agarwal et al., 2015; Shreffler et al., 2017). As a result, medically assisted reproduction (MAR), and more specifically *in vitro* fertilization (IVF) have assumed the burden of this increasing demand – these methods are becoming an increasingly more common means to conceiving a child (Sunderam et al., 2019).

While IVF is an option for many couples, rates of successful implantation and live birth hover around 50%, decreasing in chance with advancing maternal age and/or other medical complications (Fragouli et al., 2018). Typically, embryos are selected for transfer on the basis of morphology (Gardner and Schoolcraft, 1999). In the event of multiple miscarriages or advanced maternal age, patient embryos often undergo biopsy and subsequent chromosomal analysis. This procedure is referred to as PGT-A (Brezina et al., 2016). PGT-A involves the biopsy of 5–6 trophectoderm (TE) cells (comprising of approximately 3% of total TE cells) from a day-5/6 blastocyst stage embryo (Stern, 2014). Subsequent analysis of the genetic material within the cells results in identification and screening of preimplantation embryos by their ploidy status. PGT-A is known to improve pregnancy outcomes in women with recurrent pregnancy loss (Bhatt et al., 2019; Kim et al., 2019). Importantly, PGT-M/SR (preimplantation genetic testing for monogenic disorders/chromosome structural rearrangements) analysis is increasingly being utilized alongside IVF, going beyond aneuploid analysis to encompass analysis of inherited disease, single gene disorders, non-medical sexing, and structural chromosome rearrangement studies (Lathi et al., 2012; Stern, 2014). Despite significant technical capabilities clinical scientists currently employ to accurately identify genetic status (a common example being next generation sequencing), the IVF-generated euploid embryo implantation success rate remains less than 60% (Fragouli et al., 2018).

Embryo mosaicism presents potential issues in confidence of PGT-A result (Stern, 2014). In mosaic embryos cells from within the same embryo differ from one another in their genetic composition and therefore have different ploidy status. Despite the fact that 3–10 cells are biopsied for PGT-A, TE biopsy comprises ∼3% of TE cells, and >3% of total embryo cells. The differences within a single preimplantation embryo have been demonstrated and confirmed by multiple genetic methods, including microarray analysis, which illustrated the vast differences even of adjacent cells in mosaic embryos (Mertzanidou et al., 2012). Given that the frequency of mosaicism in human embryos is estimated to be approximately 20% (with a scale of 15–80% mosaicism reported within day 3 embryos), alongside evidence that aneuploidy rates decrease in the blastocyst stage of embryo development, the ability of an embryo to rid itself of aneuploid cells through regulated, and systematic means prior to implantation is a likely hypothesis (Brill et al., 1999; Mertzanidou et al., 2012; Stern, 2014). The mosaicism of a preimplantation embryo presents formidable challenges to the accuracy of PGT-A methods, even more so if an embryo has the ability to self-correct (Bolton et al., 2016).

Most recently, a randomized control trial was carried out to determine the benefits of PGT-A measured by ongoing pregnancy rate (Munné et al., 2019). This study found that PGT-A did not improve the ongoing pregnancy rate in women aged 25–40 but did improve the ongoing pregnancy rate in woman aged 35–40. These results raise the question as to why PGT-A is not improving pregnancy outcomes as expected since the now identified euploid embryo is transferred. One answer could be that there are actual mosaic preimplantation embryos that are identified as euploid by PGT-A. Similarly, there are likely other factors beyond ploidy status that allow a preimplantation embryo to successfully implant and lead to an ongoing pregnancy. Therefore, the most viable preimplantation embryo may be selected by observing a euploid ploidy status via PGT-A analysis and detection of specific proteins, mRNAs or metabolites within its blastocoel fluid. These components may reflect the preimplantation embryo’s ability and scope of self-correction prior to implantation (Brill et al., 1999; Mertzanidou et al., 2012). Obtaining the blastocoel fluid for analyses of these components is straightforward in preimplantation embryos undergoing PGT-A, as this testing requires embryo biopsy and the subsequent release of this blastocoel fluid into the surrounding media drop. Therefore, the embryo biopsy may reveal more information in addition to ploidy status (which only occurs in incidence of PGT-A) to aid the embryologist in selecting the most viable embryo for transfer. Moreover, the scope of this examination of blastocoel fluid may easily be expanded to include embryos which are frozen for subsequent transfer – frozen embryo transfer (FET).

### Blastocoel Fluid Components

**FIGURE 1:**
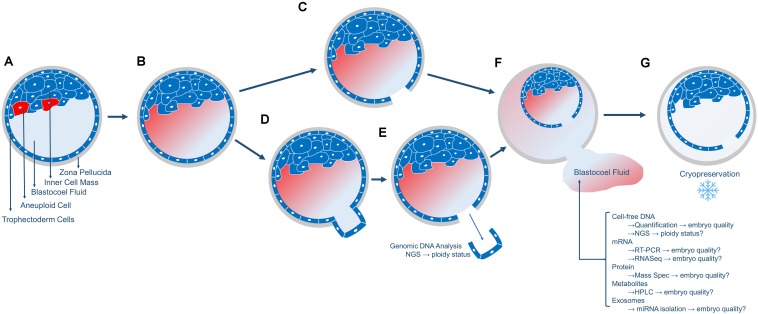
Overview of the collection of blastocoel fluid. **(A)** The day5/6 blastocyst includes the fluid-filled blastocoel cavity in contact with the inner cell mass. Red cells in the inner cell mass represent aneuploid cells that will or has undergo apoptosis. **(B)** The cells that underwent apoptosis released their contents (cell-free DNA, mRNA, proteins, metabolites, and exosomes) into the blastocoel fluid. **(C)** Blastocysts that will not undergo biopsy or embryo transfer are prepared for cryopreservation by using a laser to release the blastocoel fluid into the surrounding media drop. **(D,E)** Blastocysts that will undergo embryo biopsy for PGT-A will typically have 3–10 trophectoderm (TE) cells removed via laser-assisted cell lysis and pipette suction, with the biopsied cells sent off-site for genetic analysis. **(F)** Following either micromanipulation process, the blastocoel fluid is expelled from the blastocyst into the surrounding media and this blastocoel conditioned media can be collected and stored for subsequent analyses of the molecules as listed. **(G)** The blastocyst is cryopreserved and stored. Blastocysts are thawed once PGT-A results are known for embryo transfer.

In the case of PGT-A biopsy, a laser pulse cuts between the TE cells that have been extruded outside the zona pellucida which permits the inner blastocoel fluid to be expelled into the surrounding biopsy medium (Figures 1A–E). In the case of FET embryos (not undergoing PGT-A), prior to embryo cryopreservation, and the blastocoel fluid must be removed to prevent ice crystal formation which is accomplished via a laser pulse between TE cells (Figures 1A–C). Following blastocoel fluid expulsion, the embryo is frozen, and the blastocoel fluid-conditioned medium is routinely discarded (Figures 1F,G). Additionally, blastocoel fluid can also be obtained directly from blastocysts via blastocentesis, a minimally invasive process that uses a pipette to aspirate the blastocyst fluid directly from the blastocoel cavity and transfers it to a collection tube (Gianaroli et al., 2014). This blastocoel fluid can also be saved, frozen and assessed. Interestingly, blastocoel fluid of a preimplantation embryo is recognized as a source of cell-free DNA (cfDNA) (Capalbo et al., 2018; Li et al., 2018; Tšuiko et al., 2018; Magli et al., 2019). Please note that blastocoel fluid-conditioned biopsy media is not the same as spent culture media. Spent culture media represents embryo-conditioned growth media (i.e., released molecules during cleavage stage development).

The presence of cfDNA in human circulation was first documented in adult serum as early as 1947 and is most likely derived from circulatory cellular lysis or apoptotic activity (Swaminathan and Butt, 2006; Norwitz and Levy, 2013). In the context of human embryology, two distinct types of cfDNA exist: fetal cfDNA and postimplantation embryonic cfDNA. Postimplantation embryonic cfDNA is present within the yolk sac whereas fetal cfDNA is present within amniotic fluid and maternal circulation (Lo et al., 1997; Bianchi et al., 2001, 2014; Larrabee et al., 2004; Reece et al., 2006). Preimplantation embryonic cfDNA is a relatively recent discovery (2013) with the identification of cfDNA within the blastocoel fluid of the Day-5 human blastocyst (Palini et al., 2013). The presence of cfDNA may suggest that apoptotic activity has taken place during early embryo development (Hardy, 1999; Jurisicova and Acton, 2004). Moreover, proteins, mitochondrial DNA, and miRNAs have also been detected in the blastocoel fluid. Prior literature indicates that the origin of these molecules may potentially be remnants of cells from the developing blastocyst that underwent apoptosis during early preimplantion cell cleavage development (Palini et al., 2013; Tobler et al., 2014; Poli et al., 2015; Hammond et al., 2017; Capalbo et al., 2018). In accordance, microRNAs, some of which were linked to apoptosis, and extracellular vesicles were also found in blastocoel fluid from human preimplantation embryos (Battaglia et al., 2019). The discovery of microRNA linked to apoptosis as well as extracellular vesicles only further provide support to the existence of a preimplantation embryo self-correction mechanism. Moreover, the link to apoptosis presents a probable mechanism which potentially purges developing preimplantation embryos of aneuploid cells (Bolton et al., 2016).

Recent literature points to an increased interest in using the information provided by blastocoel cfDNA alongside PGT-A as a cumulative measure of preimplantation embryo quality (Rule et al., 2018). Several reports have postulated that competent preimplantation embryos may be identified via cfDNA content in the blastocoel fluid or spent media (Assou et al., 2014; Werner et al., 2014). This interest has recently led to increased research which analyzes the overall potential of specific cfDNA studies to reveal specific embryo insights. While some studies have reported at least a limited concordance between the chromosomal status detected using blastocoel cfDNA in comparison to PGT-A from embryonic TE biopsy, there is not enough literature or evidence to ensure that the blastocoel cfDNA analysis accurately confirms ploidy status (Ho et al., 2018; Zhang et al., 2019). The advantages of an analysis utilizing blastocoel cfDNA data rather than PGT-A are obvious (primarily the ability to test for aneuploidy without performing embryo biopsy), but the theoretical concept remains unproven, as the pragmatic concordance has yet to reach a satisfactory level. A truly non-invasive approach to assess preimplantation embryo ploidy status (non-invasive PGT-A) would involve analysis of spent media from preimplantation embryos cultured from early cell cleavage stages to the blastocyst stage that did not undergo embryo biopsy. A proof-of-concept study thawed and cultured previously frozen donated blastocysts (with known ploidy status from embryo biopsy) and then collected their spent media, which likely contained cfDNA from blastocoel fluid (Huang et al., 2019). The spent media was then assessed for PGT-A and revealed high concordance with the PGT-A results from the TE biopsy. Though this study suggests that non-invasive PGT-A from spent media is promising, the manner in which the media was collected in the study is not a routine procedure for IVF cases. However, combining PGT-A from TE biopsy with embryonic cfDNA analysis (DNA obtained from spent blastocyst medium) has improved implantation rates (Rubio et al., 2019) and additional analyses on blastocoel components may further enhance implantation rates.

### Potential Applications of Blastocoel Components

While the application of cfDNA analysis from spent media as a replacement for PGT-A is problematic and risky at best, there may yet be means through which cfDNA can provide critical information about a preimplantation embryo alongside PGT-A. In our lab’s research, we have observed that the quantitative analysis of cfDNA content have been directly correlated to both preimplantation embryo morphology and ploidy status (Rule et al., 2018; Chosed et al., 2019). Since it has been reported that the quantity of cfDNA is correlated with ploidy status, one way to utilize the blastocoel fluid would involve measuring cfDNA quantity as a confirmational check to any PGT-A result, potentially providing a second determinant of embryo quality (Chosed et al., 2019). As demonstrated through example, this blastocoel fluid-derived correlation may be of use to provide additional data points and guidance for embryo transfer selection beyond results from PGT-A.

Since cfDNA and other molecular remnants in the blastocoel fluid likely originate from apoptosis occurring within the preimplantation embryo, identification, and analysis of these molecules may also provide insight into the self-correction status of the blastocyst. mRNAs expressed during the apoptotic program in the developing embryo also reside within the blastocoel fluid (Athavale et al., 2019; Barré et al., 2019; Kranyak et al., 2019). Differences in expression of genes may be detected in blastocoel fluid from preimplantation embryos with differing ploidy status or implantation potential. It is therefore possible to analyze blastocoel fluid to provide further information regarding the viability of an embryo. Battaglia et al., have identified extracellular vesicles harboring microRNAs within the blastocoel fluid, therefore these molecules offer yet another source of information that may provide insight into the viability of the preimplantation euploid embryo. A more focused analysis of blastocoel components and/or spent media will provide clues as to what these molecules represent and will provide an unprecedented opportunity to uncover the development processes that ensures a viable preimplantation embryo is available for a successful implantation.

## Discussion

As MAR continues to be practiced around the world, there is a growing need for developing methods which assist in maximizing probability of implantation while minimizing cycle count. Analyzing the blastocoel fluid components can provide the clinician another measure in addition to preimplantation embryo morphology and ploidy status obtained from PGT-A. Furthermore, a more thorough analysis may lead to additional insight regarding the self-correction mechanisms of embryos, or the implantation potential of specific preimplantation embryos. The study of blastocoel fluid components can provide a clinician with a greater understanding of the preimplantation embryo’s overall potential to generate a live-birth. Overall, given the ability to provide further information to current embryo quality tests with no additional interference to embryo growth, we argue that study of additional molecular information obtained from the blastocoel fluid at the time of biopsy which may aid in selection of the most viable preimplantation embryo for transfer warrants further analysis and eventual clinical application.

## Author Contributions

AL led manuscript writing with assistance from WR and RC. All authors contributed to the drafting process.

## Conflict of Interest

The authors declare that the research was conducted in the absence of any commercial or financial relationships that could be construed as a potential conflict of interest.
